# Effect of Crude Polysaccharides from *Ecklonia cava* Hydrolysate on Cell Proliferation and Differentiation of *Hanwoo* Muscle Stem Cells for Cultured Meat Production

**DOI:** 10.3390/foods13040563

**Published:** 2024-02-13

**Authors:** Jae-Hoon Lee, Tae-Kyung Kim, Min-Cheol Kang, Min-Kyung Park, Sang-Hun Park, Jung-Seok Choi, Yun-Sang Choi

**Affiliations:** 1 Research Group of Food Processing, Korea Food Research Institute, Wanju 55365, Republic of Korea; l.jaehoon@kfri.re.kr (J.-H.L.); ktk.kim@kfri.re.kr (T.-K.K.); mckang@kfri.re.kr (M.-C.K.); mk.park@kfri.re.kr (M.-K.P.); 2 Department of Animal Science, Chungbuk National University, Cheonju 28644, Republic of Korea

**Keywords:** *Ecklonia cava*, polysaccharide, proliferation, differentiation, *Hanwoo* muscle satellite cells

## Abstract

*Ecklonia cava*, a brown seaweed native to the East Asian coast, is known for its unique composition, including polysaccharides, polyphenols, and phlorotannins. Fucoidan is a sulfated polysaccharide widely used as a functional ingredient in foods. This study obtained crude polysaccharides (ECC_CPS) from *E. cava* celluclast enzymatic hydrolysate using ethanol precipitation. ECC_CPS increased cell viability during the proliferation of *Hanwoo* muscle satellite cells (HMSCs). The effect of ECC_CPS on the expression of proliferation-related markers was confirmed as *MYF5* and *MYOD* expression significantly increased, whereas *PAX7* expression was maintained. The evaluation of cell migration activity has a major impact on cell proliferation and differentiation, and the cell migration index significantly increased with ECC_CPS treatment (*p* < 0.01). This was related to the HGF/MET pathway and FAK pathway. Treatment with ECC_CPS promoted differentiation at the cell differentiation stage, thereby increasing the expression of differentiation markers, such as *MYH2*, *MYH7*, and *MYOG* (*p* < 0.001 or *p* < 0.01). Therefore, our findings imply that crude polysaccharide obtained from E. cava can be an additive ingredient that enhances the proliferation and differentiation of muscle satellite cells used in the manufacture of cultured meat products.

## 1. Introduction

The world population is predicted to reach 10 billion by 2050, and food shortage problems will occur solely because of the increase in protein production through traditional livestock farming, which relies on livestock raising [1]. Food security is becoming an increasingly critical issue in light of the global food crisis and increasing environmental pollution [2]. To ensure food security, national support is being provided for international cell-culture-based food research, and various measures are being implemented for exploring alternative food sources [3]. Research on cell-culture-based meat is in its early stages, and the market has not yet been formed; therefore, the foundations of the food-tech industry are currently insufficient for securing food sovereignty [4,5].

With increasing concerns about climate change, carbon footprints are being increasingly investigated, with food systems being one of the major contributors [6]. Among the various livestock species, beef cattle have been recognized as major contributors to greenhouse gas emissions, primarily due to enteric fermentation [7]. Raising beef cattle can thus result in substantial greenhouse gas production and environmental pollution. Consequently, continually increasing the population of beef cattle is not feasible.

The sustainability of food systems is crucial for present and future generations. Therefore, the production of artificial meat using cellular agriculture has emerged as a novel sustainable protein production method [8]. One piece of muscle tissue can be multiplied by a trillion strands, which can easily alleviate problems of protein scarcity [9]. Cell proliferation and differentiation must be enhanced to produce large quantities of meat [10]. The activation of satellite cells should be improved to increase cell proliferation, provide abundant progeny, and support the generation of muscle tissue [11].

Seaweeds have long been known for their nutritional richness and diverse bioactive constituents [12]. *Ecklonia cava*, a brown alga native to East Asian coastal regions, is known for its unique composition, encompassing polysaccharides, polyphenols, phlorotannins, and other bioactive molecules [13]. Several studies have demonstrated that these compounds have antioxidant, anti-inflammatory, and antimicrobial properties [14]. Importantly, recent studies have suggested their potential role in modulating cellular processes, specifically cell proliferation and differentiation [15,16,17,18]. If hydrolyzed *E. cava* extract and its crude polysaccharide are proven to be effective in promoting muscle cell proliferation and differentiation in *Hanwoo*, they could serve as a valuable and eco-friendly supplements in the production of cultured meat using *Hanwoo* muscle cells.

Therefore, the objective of this study was to comprehensively evaluate the effect of hydrolyzed *E. cava* extract and its crude polysaccharide on the proliferation and differentiation of *Hanwoo* muscle cells, with a specific emphasis on proliferation and differentiation of the satellite cells.

## 2. Materials and Methods

### 2.1. Hydrolysis of E. cava and Preparation of Crude Polysaccharide

*Ecklonia cava* was purchased from a local market on Jeju Island (Korea), washed three times, freeze-dried, and ground into powder. Enzymatic hydrolysis was performed using the glycolytic enzyme celluclast (Novozymes, Bagsvaerd, Denmark). Next, 3 g of *E. cava* powder was suspended in distilled water and incubated with 30 mg of celluclast (Enzyme–substrate = 1:100) under the conditions of a shaking incubator (pH 4.5, 50 °C, 120 rpm, and 24 h). The enzyme was then inactivated by heating at 100 °C for 10 min. The hydrolysate was centrifuged at 13,000× *g* for 10 min at 4 °C. The pH of the supernatant was adjusted to 7.0 using NaOH (1 M, Sigma-Aldrich, St. Louis, MO, USA)). The celluclast enzyme hydrolysate of *E. cava* was named ECC.

Crude polysaccharides from ECC were obtained via gradient ethanol precipitation [19]. Briefly, 99.5% ethanol was mixed with ECC (1 L:0.5 L), and then the crude polysaccharide was precipitated for 12 h at 4 °C. Centrifugation was performed at 5000× *g* for 20 min, and the precipitate was named ECC_CPS1. This process was repeated to obtain ECC_CPS2 and ECC_CPS3. Finally, the ECC and ECC_CPS were freeze-dried and used in future studies.

### 2.2. Monosaccharide Composition Analysis Using High-Performance Anion-Exchange Chromatography

The monosaccharide composition of ECC and ECC_CPSs was analyzed using high-performance anion-exchange chromatography (HPAEC) [20]. The used detector and column were pulsed amperometric detector (Dionex, Sunnyvale, CA, USA) and CarboPac™ PA10 column (I.D.: 2 mm, length: 250 mm, particle size: 10 μm, Dionex) for separation. All ECC and ECC_CPSs were hydrolyzed using trifluoroacetic acid (Sigma-Aldrich). The injection volume was 20 μL, flow rate was 1 mL/min, and eluent A/B was 18 mM NaOH/200 mM NaOH. The eluent program was as follows: 0–20 min, 0% B; 20–35 min, 100% B; 35–45 min, 0% B. 

### 2.3. Primary Hanwoo Muscle Satellite Cell Isolation (HMSCs)

Briskets from 34-month-old castrated *Hanwoo* steers were collected from slaughterhouse A in Eumseong-gun, Chungcheongbuk-do, Republic of Korea. After storage in an icebox and transportation to the laboratory, muscle tissue was collected and treated with a collagenase type II mix (Worthington, Lakewood, NJ, USA). Connective tissues and muscle cells were separated by repeated centrifugation at 70× *g* and 800× *g*. The pellet (muscle cells) obtained using centrifugation was filtered through 100 μm and 40 μm filters and red blood cells were lysed with Ammonium–Chloride–Potassium (ACK) lysis buffer (ThermoFisher, Waltham, MA, USA). The extracted muscle cells were placed in a freezing medium (Gibco, Grand Island, NY, USA) and stored in liquid nitrogen until analysis.

Prior to purification of *Hanwoo* muscle satellite cells (HMSCs) using fluorescence activated cell sorting (FACS), the extracted *Hanwoo* muscle cells were cultured in flasks coated with 5 mg/mL bovine collagen type I (Sigma-Aldrich) until confluent. Cells were suspended in a FACS buffer (1:100, bovine serum albumin in phosphate-buffered saline (PBS)) and incubated with APC anti-human CD29 antibody (1:10, BioLegend, San Diego, CA, USA), PE-CyTM7 anti-human CD56 antibody (1:10, BD Biosciences, Lakes, NJ, USA), FITC anti-sheep CD31 antibody (1:10, Bio-Rad, Hercules, CA, USA), and FITC anti-sheep CD45 antibody (1:10, Bio-Rad) for 30 min at 4 °C [21]. After antibody treatment, cells were washed twice with cold PBS and resuspended in FACS buffer. CD31^-^, CD45^-^, CD29^+^, and CD56^+^ cells were sorted using a FACS Aria II Cell Sorter (BD Biosciences).

### 2.4. Cell Proliferation and Differentiation

Cell proliferation was assessed using Ham’s F-10 medium (Gibco) containing 20% fetal bovine serum (FBS, Gibco), 1% antibiotic mixture (Lonza, Morrisville, NC, USA), and 0.05% basic fibroblast growth factor (bFGF, Gibco). Cell differentiation was performed in Dulbecco’s Modified Eagle Medium (DMEM) (Gibco) containing 2% FBS and 1% antibiotic mixture. Cell proliferation cultures were seeded at 1800/cm^2^ and cultured in a humidified CO_2_ incubator at 37 °C, 5% CO_2_ with Ham’s F-10 media for 7 d. Cell differentiation cultures were seeded at 5000/cm^2^ and cultured under the same conditions as the proliferation cultures, and Ham’s F-10 medium was replaced with DMEM when the cells reached confluency in 80–90% of the area in the flask. Proliferated HMSCs were observed using an EVOS M5000 imaging system (ThermoFisher) at the same time each day.

### 2.5. Cell Proliferation Assay

HMSC proliferation was measured using the CellTiter 96^®^ Proliferation Assay (Promega, Madison, WI, USA). HMSCs were cultured in 96-well plates for 4 d with ECC and ECC_CPSs (10, 50, and 100 μg/mL), and the assay uses the principle that 3-(4,5-dimethylthiazol-2-yl)-5-(3-carboxymethoxyphenyl)-2-(4-sulfophenyl)-2H-tetrazolium (MTS) is converted to formazan by the dehydrogenase enzyme in the cells. MTS reagent was dispensed into each well and further incubated for 2 h, and the absorbance was measured at 490 nm.

### 2.6. Quantitative Real-Time PCR (qRT-PCR)

The extraction of mRNA from HMSCs cultured for 4 d with ECC_CPS3 was performed according to the manufacturer’s manual of the Total RNA Extraction Kit (iNtRON Biotechnology, Seongnam, Korea). Extracted mRNA was used to prepare cDNA according to the manufacturer’s instructions for the cDNA Reverse Transcription Kit (ThermoFisher). PCR was performed using SYBR Green (ELPIS-BIOTECH, Daejeon, Republic of Korea) and the primer sequences listed in Table 1. Gene amplification was as follows: 50 °C for 2 min, 95 °C for 10 min, followed by 40 cycles of 95 °C for 10 s and 20 s between 52 and 55 °C. The mRNA quantification method used the 2^−ΔΔCT^ method [22].

### 2.7. Cell Migration Effect (Wound-Healing Assay)

To determine cell migration, a wound-healing assay was performed. HMSCs were incubated in 6-well plates with ECC_CPS3. The next day, confluent cells were scraped evenly across the wells using a 1 mL pipette tip. The plates were incubated for 24 h. The scratched regions were photographed at 0 and 24 h using an EVOS-M5000 imaging system. The migration index was analyzed using ImageJ software (for Window ver., NIH, Bethesda, MD, USA).

### 2.8. Statistical Analysis

To test the significance of the results (*n* = 3), the statistical processing program SPSS (ver. 20, SPSS Inc., Chicago, IL, USA). To compare significant differences between measurements, Student’s *t*-test or Duncan’s multiple range test was performed (*p* < 0.05).

## 3. Results and Discussion

### 3.1. Monosaccharide Composition Analysis of E. cava Extract

**Figure 1:**
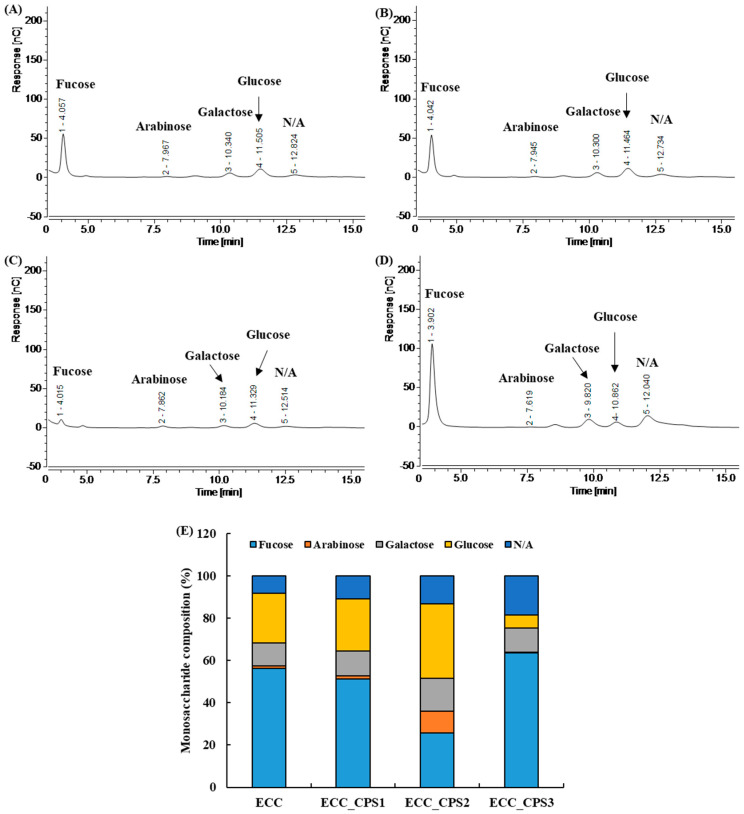
Monosaccharide composition analysis: HPAEC chromatogram of ECC, ECC_CPS1, ECC_CPS2, and ECC_CPS3 (**A**–**D**), composition ratio (**E**). N/A means not available. ECC, *E. cava* celluclast enzyme extract; ECC_CPS, crude polysaccharide extracts of ECC.

The functional activities of polysaccharides are greatly influenced by the monosaccharides that they contain [20]. Therefore, the monosaccharide compositions of ECC and ECC_CPSs as revealed by HPAEC analysis are shown in Figure 1.

ECC and ECC_CPSs are composed of four major monosaccharides: fucose, arabinose, galactose, and glucose. Among the four monosaccharides, fucose was the most abundant (ECC: 56.13%; ECC_CPS1: 51.17%; ECC_CPS3: 63.66%). However, for ECC_CPS2, the glucose content (35.03%) was higher than the fucose content (25.82%). ECC_CPS3 had the highest fucose content, and as a result, its glucose content was the lowest compared to the other samples (ECC: 23.44%; ECC_CPS1: 24.65%; ECC_CPS2: 35.03%; ECC_CPS3: 6.12%). In the monosaccharide analysis of the crude polysaccharide, the high fucose content suggests that the content of fucoidan, a sulfated polysaccharide, may be high. Previous studies have reported a high fucoidan content in the polysaccharide extracts of *E. cava* [23]. Since fucoidan has been reported to have high antioxidant activity, it is expected to have a positive effect on various biological functions in cells.

The cell-proliferative effects of polysaccharides are obtained from various materials [24,25]. Polysaccharides extracted from *Ganoderma amboinense*, a type of mushroom, have a proliferative effect on normal cells (293T/17, HUV-EC-C, NIH/3T3, and THP-1) [25]. Additionally, Yang et al. [24] reported that polysaccharides obtained from *Usnea longissima* (moss) via ethanol precipitation significantly increased the proliferation of human HaCaT keratinocytes. In these studies, the authors explained that the antioxidant activity of polysaccharides had a positive effect on cell proliferation. Therefore, considering the monosaccharide composition results of ECC and ECC_CPSs (Figure 1) and preliminary research reports showing a high fucoidan content [23], ECC and ECC_CPS are expected to have positive effects on the proliferation and differentiation of MSCs.

### 3.2. Effect of E. cava Extract on Proliferation Capacity of HMSCs

**Figure 2:**
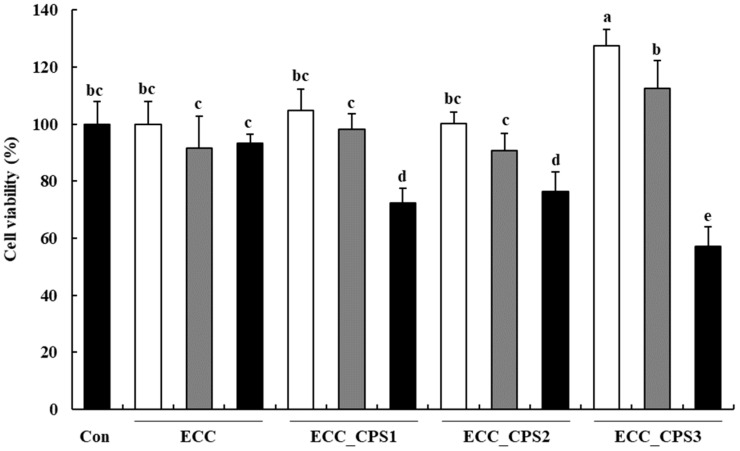
Effects of ECC and ECC_CPSs (10, 50, and 100 μg/mL) on the cell viability of HMSCs. White, grey, and black bars represent 10, 50, and 100 μg/mL concentrations. All experimental data are expressed as the mean ± SD. Different letters (a–e) indicate significant differences (*p* < 0.05). Con, medium-only treated group; ECC, *E. cava* celluclast enzyme extract; ECC_CPS, crude polysaccharide extracts of ECC.

The cell proliferation capacity as determined using the MTS assay, to investigate the effect of *E. cava* celluclast enzyme extract (ECC) and its crude polysaccharide extracts (ECC_CPS1, ECC_CPS2, and ECC_CPS3) on the proliferation of HMSCs, after 4 d of culture is shown in Figure 2. In the case of ECC, in which the crude polysaccharide was not separated through ethanol precipitation, no significant changes in cell viability compared to the control were observed at all tested concentrations (*p* > 0.05). ECC_CPS1 and ECC_CPS2 showed no significant differences in cell viability; therefore, their proliferative effect on HMSCs could not be confirmed. However, treatment with high concentrations (100 μg/mL) of ECC_CPS1 and ECC_CPS2 showed a lower cell viability compared to the control (*p* < 0.05), while high concentrations showed cytotoxicity. In contrast, ECC_CPS3 showed significantly higher cell viability than the control (10 μg/mL, *p* < 0.05). Thus, ECC_CPS3 exerted a proliferative effect on HMSCs. However, at a high concentration of 100 μg/mL, it showed cytotoxicity similar to the other CPS samples.

**Figure 3:**
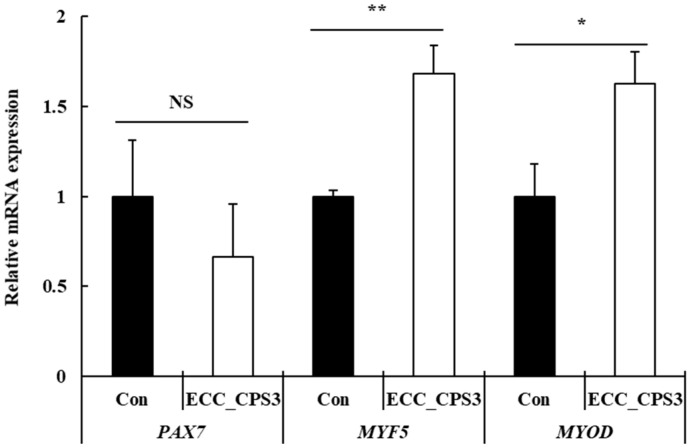
Effects of ECC_CPS3 (10 μg/mL) on mRNA expression of proliferation markers (*PAX7, MYF5* and *MYOD*) in HMSCs. All experimental data were expressed as the mean ± SD. * *p* < 0.05, ** *p* < 0.01 considered statistically significant between ECC_CPS3 and Con. NS indicates no significant difference between ECC_CPS3 and Con (*p* > 0.05). Con, medium-only treated group; ECC_CPS, Crude polysaccharide extracts of *E. cava* celluclast enzyme extract.

As shown in Figure 3, the analysis of the mRNA expression of cell proliferation markers (*PAX7, MYF5,* and *MYOD*) revealed that ECC_CPS3 significantly enhanced the expression of *MYF5* (*p* < 0.01) and *MYOD* (*p* < 0.05) among cell proliferation markers. There was no significant difference in *PAX7* expression in the control group (*p* > 0.05). *PAX7* is a myogenic marker predominantly expressed in quiescent satellite cells and plays a major role in maintaining self-renewal, a key characteristic of stem cell stemness [26]. Additionally, a decrease in *PAX7* expression induces cell cycle arrest, leading to the differentiation of muscle stem cells [27]. In this study, we confirmed that *PAX7* expression in HMSCs was maintained following ECC_CPS3 treatment (Figure 3). This finding indicates that ECC_CPS3 can preserve the self-renewal capacity of HMSCs and is believed to promote proliferation in the proliferation phase rather than moving to the differentiation phase. *MYF5* is expressed when quiescent satellite cells are activated and begin to proliferate into myoblasts [28]. Subsequently, *MYOD* is expressed, which regulates myoblast commitment [29]. *MYF5* and *MYOD* belong to the myogenic regulatory factor family (MRF) and can convert various differentiated cell types to myogenesis [30]. In this study, *MYF5* and *MYOD* expression were increased by treatment with ECC_CPS3 (Figure 3). An increase in *MYF5* expression plays a major role in the activation of satellite cells and promotes the initiation of cell proliferation, whereas an increase in *MYOD* expression can cause satellite cells to lose their proliferative capacity, stop proliferating, and move to the differentiation phase [31]. However, in this study, with ECC_CPS3 treatment, the expression of *PAX7* was maintained rather than decreased along with an increase in *MYF5* and *MYOD*, which means that the proliferation capacity did not decrease [32]. Kim et al. [28] reported the effect of treatment with fermented soybean meal and edible insect protein hydrolysate on the proliferation markers of pig muscle stem cells in a medium with low FBS content. Kim et al. [28] reported that among the proliferation markers, the expression of *MYF5* and *MYOD* significantly increased, while the expression of PAX7 was maintained, thereby indicating that the proliferative potential of pig muscle stem cells is high.

### 3.3. Effect of E. cava on Cell Migration Effect of HMSCs 

**Figure 4:**
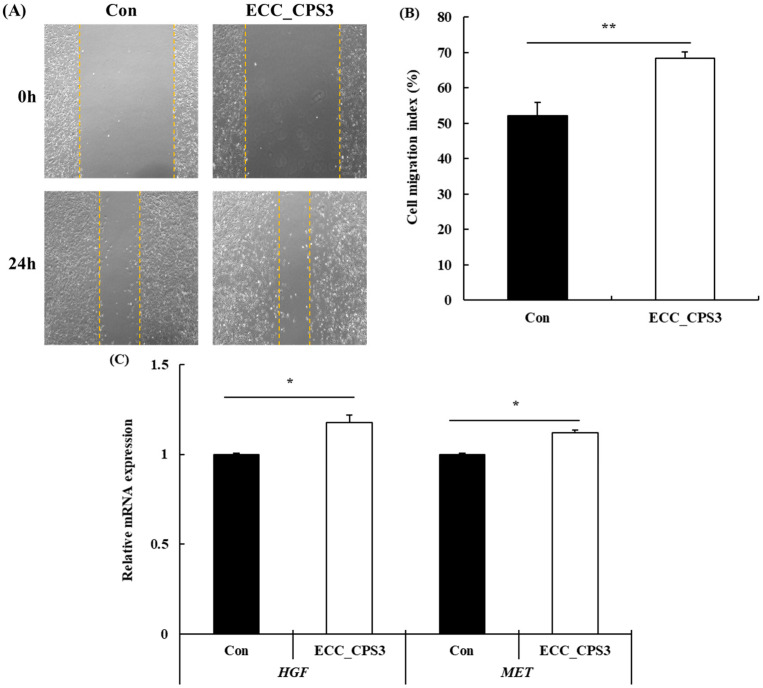
Effects of ECC_CPS3 (10 μg/mL) on the cell migration activity of HMSCs: wound-healing images (**A**), cell migration index (%) (**B**), and mRNA expression of cell migration markers (*HGF* and *MET*) (**C**). All experimental data are expressed as the mean ± SD. * *p* < 0.05, ** *p* < 0.01 considered statistically significant between ECC_CPS3 and Con. Con, medium-only treated group; ECC_CPS, Crude polysaccharide extracts of *E. cava* celluclast enzyme extract. Yellow lines indicate cell migration range.

Cell migration plays a crucial role in various biological functions of cells (e.g., muscle differentiation, development, and regeneration) [33]. Therefore, to confirm the effect of ECC_CPS3 on the migration of HMSCs, a wound-healing assay was conducted, and the effect on mRNA expression related to cell migration was confirmed (Figure 4).

Based on the cell migration index (%), calculated by comparing the scratch distance between 0 and 24 h, the migration index of the control was 52.05% (Figure 4B). In contrast, the treatment group with ECC_CPS3 showed a migration index of 68.47%, confirming a significant increase in cell migration (*p* < 0.01). This finding suggests that treatment with ECC_CPS3 increases the migratory activity of HMSCs, ultimately affecting cell proliferation and differentiation. The positive effect of ECC_CPS3 on cell migration was confirmed by analyzing the expression of cell-migration-related mRNAs (Figure 4C). Treatment with ECC_CPS3 significantly increased the expression of the hepatocyte growth factor (*HGF*) and mesenchymal–epithelial transition factor (*MET*) in HMSCs compared to the control group (*p* < 0.05).

HGF is required for the activation of muscle satellite cells and plays a major role in myoblast migration [34]. MET is a receptor (HGFR) for HGF [35]. MET is a tyrosine kinase receptor that triggers cellular biological activity by binding to HGF. These processes include proliferation, differentiation, survival, motility, and extracellular matrix (ECM) degradation [36]. MET plays a major role in the regulation of myoblast migration and fusion [37]. Therefore, *HGF* and *MET* and their expressions can be used as positive markers of cell migration. In this study, treatment with ECC_CPS3 enhanced the expression of *HGF* and *MET* in HMSCs, and the cell migration index increased, which indicates a cell migration effect.

**Figure 5:**
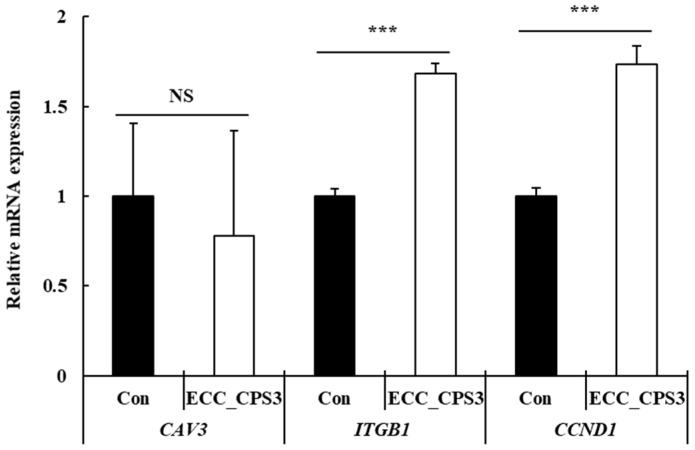
Effects of ECC_CPS3 (10 μg/mL) on focal adhesion kinase (FAK)-related mRNA expression (*CAV3*, *ITGB1*, and *CCND1*) in HMSCs. All experimental data are expressed as the mean ± SD. *** *p* < 0.001 is considered statistically significant between ECC_CPS3 and Con. NS indicates no significant difference between ECC_CPS3 and Con (*p* > 0.05). Con, medium-only treated group; ECC_CPS, crude polysaccharide extracts of *E. cava* celluclast enzyme extract.

To further understand the effect of ECC_CPS3 on cell migration, we determined the effect of ECC_CPS3 on the mRNA expression levels of focal adhesion kinase (FAK) signaling pathway markers (Figure 5). ECC_CPS3 treatment significantly increased the expressions of *ITGB1* and *CCND1* in HMSCs (*p* < 0.001). However, there was no significant effect on *CAV3* expression (*p* > 0.05). The FAK signaling pathway is a non-receptor tyrosine kinase that plays a role in cell migration and adhesion and is important for muscle wound healing and regeneration [38]. Activated FAK combines with integrins in the cell membrane to form focal adhesions and plays a role in cell adhesion and migration [39]. Therefore, the expression of integrin subunit β1 (*IGTB1*) and cyclin D1 (*CCND1*) following ECC_CPS3 treatment indicates the activation of the FAK signaling pathway and is believed to be related to the cell-migration-enhancing effect of ECC_CPS3. This enhanced cell migration activity is thought to positively affect the proliferation and differentiation of HMSCs.

### 3.4. Effect of E. cava Extract on Differentiation Capacity of HMSCs 

**Figure 6:**
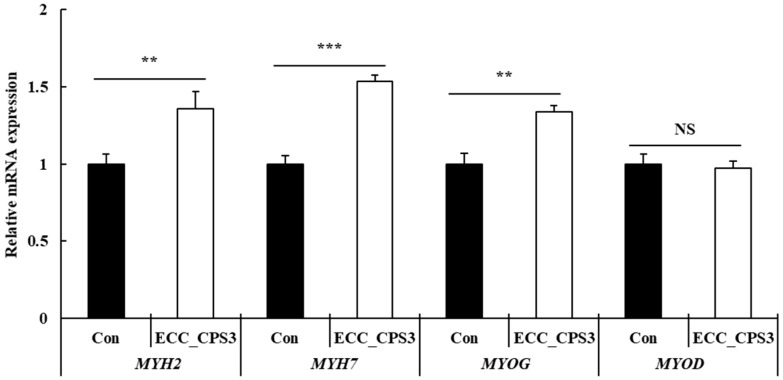
Effects of ECC_CPS3 (10 μg/mL) on mRNA expression of differentiation markers (*MYH2, MYH7*, *MYOG*, and *MYOD*) in HMSCs. All experimental data are expressed as the mean ± SD. ** *p* < 0.01, *** *p* < 0.001 are considered statistically significant between ECC_CPS3 and Con. NS indicates no significant difference between ECC_CPS3 and Con (*p* > 0.05). Con, medium-only treated group; ECC_CPS, Crude polysaccharide extracts of *E. cava* celluclast enzyme extract.

As shown in Figure 6, treatment with ECC_CPS3 resulted in a significant increase in the mRNA levels of cell differentiation markers, such as *MYH2* (*p* < 0.01)*, MYH7* (*p* < 0.001)*,* and *MYOG* (*p* < 0.01), compared to the control group, as revealed using qRT-PCR analysis conducted on the 4th day of differentiation. In contrast, ECC_CPS3 had no significant effect on *MYOD* mRNA expression (*p* > 0.05).

Myogenin (*MYOG*) is a member of the MRF family [40]. It is the main target of *MYOD* and plays a role in initiating terminal differentiation along with MRF4. It is involved in key reactions during myogenesis, including myoblast-to-myocyte transition, myotube fusion, and myofiber maturation [41]. Furthermore, the myosin heavy chain (MYHC) is one of the representative late-stage differentiation markers whose expression level increases as muscle cell differentiation progresses [22]. *MYH2* and *MYH7* are known expression factors that are used to identify myofiber types IIa and I, respectively [28]. Treatment with ECC_CPS3 increased the expression of *MYOG* by 1.34-fold and the expressions of *MYH2* and *MYH7* by 1.36-fold and 1.54-fold, respectively (Figure 6). The expressions of *MYOG* and *MYH*s indicate the early and late stages of differentiation, respectively, and the expression of *MYH7* is significantly higher than that of *MYOG* [42], implying that ECC_CPS3 treatment had an excellent ability to promote differentiation and that sufficient differentiation had already occurred on the fourth day of differentiation. In contrast, the expression of *MYOD* did not significantly change after ECC_CPS3 treatment at the differentiation stage (*p* > 0.05). This result was contrary to the finding that treatment with ECC_CPS3 significantly increased the expression of *MYOD* at the proliferation stage (Figure 3). *MYOD*, together with *MYF5*, is used as a marker for undifferentiated proliferating myoblasts and *MYOD* plays a role in initiating differentiation at the myoblast stage before differentiation [43]. Therefore, we believe that the expression of *MYOD* was not affected by ECC_CPS3 treatment when differentiation had already begun on the 4th day.

Research is actively underway to find substances in natural products that can promote the differentiation of muscle stem cells. Guo et al. [15] reported that three flavonoids (quercetin, icariin, and 3,2′-dihydroxyflavone) exhibited pro-differentiation effects by increasing the expression of *MYHC* mRNA in porcine muscle stem cells. The degree of cell differentiation into multinucleated myotubes was confirmed by determining *MYHC* mRNA expression. In addition, the effect of *Tenebrio molitor* enzyme hydrolysates (Alcalase and Flavourzyme), commonly known as mealworms, on the differentiation of porcine muscle stem cells has been reported [28]. The expressions of *MYH1* and *MYH4*, which are cell differentiation markers, significantly increased, and myotube formation was better than that of the control group in cell morphology after 48 h of differentiation [28]. Additionally, it has been reported that polysaccharide extracted from *Bletilla striata* enhances human tenocyte (a type of connective tissue cell that connects muscles to bone) proliferation [17]. The study reported that polysaccharide had the effect of promoting proliferation by activating various signaling pathways, such as MEK/ERK1/2 and PI3K/Akt.

Many studies on the functionality of crude polysaccharide obtained from seaweed through ethanol precipitation have already been reported [20,44,45]. It can be confirmed through previous studies that the content of polysaccharide in sediment increases during ethanol precipitation [20,45,46]. Therefore, it is thought that the positive effect of ECC_CPS3 on the proliferation and differentiation of muscle stem cells obtained from this study was caused by the polysaccharide present in the *E. cava* extract.

## 4. Conclusions

In conclusion, we found that crude polysaccharides obtained from *E. cava* celluclast enzymatic hydrolysates can facilitate the proliferation and differentiation of *Hanwoo* muscle satellite cells (HMSCs). Treatment with ECC_CPS3 obtained through ethanol precipitation significantly increased the viability of HMSCs. In addition, it significantly increased the expression of cell proliferation markers *MYF5* and *MYOD* mRNA and maintained the expression of *PAX7*, thereby confirming that it maintained the stemness of HMSCs and promoted proliferation. We confirmed that the cell migration activity of HMSCs was increased by activating the HGF/MET and FAK pathways related to cell migration. Finally, differentiation was promoted at the cell differentiation stage, enhancing the expression of differentiation markers, such as *MYH2*, *MYH7*, and *MYOG*. These findings suggest that crude polysaccharide obtained from *Ecklonia cava* is highly promising as an additive that can efficiently promote proliferation and differentiation in the production of cultured meat derived from *Hanwoo*.

## Figures and Tables

**Table 1:** Primer sequences used in RT-qPCR analysis.

Primer	Description	Sequence (5′-3′)
PAX7	Paired box 7	F: AGCCGGGTTAGCAAGATACT R: GAAACTCTGGCTGACCTTGA
MYF5	Myogenic factor 5	F: ATTACCAGAGACACCGACCA R: CAGGAGCCGTCGTAGAAGTA
MYOD	Myoblast determination protein 1	F: AACAGCGGACGACTTCTATG R: GTTAGTCGTCTTGCGTTTGC
HGF	Hepatocyte growth factor	F: CACACGAACACAGCTTTTTG R: ATGGGACCTCGGTAACTTTC
MET	Mesenchymal–epithelial transition factor	F: TTCATTGGGGAGCACTATGT R: CAAAGGGTGGACTGTTGTTC
CAV3	Cavolin 3	F: AAAGACAGTCCACCATGGAA R: ACCGAAACCCTTTATTGGAG
ITGB1	Integrin subunit β1	F: AGATGAGGTGAACAGCGAAG R: CTCACACACTCGACACTTGC
CCND1	Cyclin D1	F: CTCGAAGATGAAGGAGACCA R: GAAGTGCTCGATGAAGTCGT
MYH2	Myosin heavy chain 2	F: CCAGTGGAGGACCAAGTATG R: TTCCTTTGCTTTTTGTCCAG
MYH7	Myosin heavy chain 7	F: TGGACAAGAAGCAGAGGAAC R: TTGAAGGTCTCCAGATGCTC
MYOG	Myogenin	F: ACAAACCATGCACATCTCCT R: AGCACAGAGACCTTGGTCAG
β-Actin		F: CGCAGAAAACGAGATGAGAT R: CTCGGCCACACTGTAGAACT

## Data Availability

The original contributions presented in the study are included in the article, further inquiries can be directed to the corresponding authors.
